# Iron metabolism patterns in non-anemic patients with myasthenia gravis: A cross-sectional and follow-up study

**DOI:** 10.3389/fneur.2022.1060204

**Published:** 2022-11-24

**Authors:** Ke Li, Li'an Hou, Ying Tan, Yangyu Huang, Jiayu Shi, Jianhua Han, Jingwen Yan, Yuzhou Guan

**Affiliations:** ^1^Department of Neurology, Peking Union Medical College Hospital, Chinese Academy of Medical Sciences and Peking Union Medical College, Beijing, China; ^2^Department of Laboratory Medicine, Peking Union Medical College Hospital, Chinese Academy of Medical Sciences and Peking Union Medical College, Beijing, China

**Keywords:** myasthenia gravis, autoimmune diseases, iron metabolism disorders, iron deficiency, cross-sectional studies, follow-up

## Abstract

**Background and purpose:**

Iron metabolism in myasthenia gravis (MG) and factors associated with it are explored by few published studies. Therefore, this study aimed to compare iron metabolism patterns between patients with MG and healthy individuals as well as between the same group of patients before and after immunotherapy, and to identify predictors of iron metabolism disorders in MG.

**Materials and methods:**

For this study, 105 patients and healthy individuals were included at baseline, after which paired parametric and non-parametric tests were adopted to compare their iron metabolism patterns, and multivariate binary logistic regression was used to identify predictors of iron metabolism disorders. Patients with MG were then followed up for 12 ± 3 months to explore alterations in their iron metabolism patterns after starting immunotherapy with the help of paired tests.

**Results:**

Non-anemic immunotherapy-naive patients with MG had significantly lower serum iron (SI) and transferrin saturation (TS) levels than healthy individuals. Premenopausal female was significantly associated with SI < 65 μg/dL and iron deficiency in these patients. However, iron metabolism parameters did not significantly alter after around 12 months of immunotherapy in patients with MG.

**Conclusion:**

Iron inadequacy was present in patients with MG, particularly premenopausal female patients, and it would hardly improve after immunotherapy. Given the significant role of iron in human body, it should be given more attention in patients with MG.

## Introduction

Myasthenia gravis (MG) is an autoimmune disease resulting from impairment of the postsynaptic membrane at the neuromuscular junction and mostly manifests as skeletal muscle fatigue. The prevalence of MG was estimated to be 64.0–94.3 cases per million (1). In MG, two of the most common pathogenic autoantibodies are anti-acetylcholine receptor antibody (AChR-Ab) and anti-muscle-specific tyrosine kinase antibody (MuSK-Ab).

Iron is an essential element for physiological and pathophysiological processes in living organisms, involving in autoimmunity (2), tumorigenesis (3), and infection (4). In the human nervous system, iron plays important roles in myelination, neurotransmitter synthesis, oxidative phosphorylation, and other crucial processes (5). Recent evidence has indicated that iron metabolism disorders might be prominent in neuroimmune diseases, including multiple sclerosis (5–7), polymyositis, and dermatomyositis (8–10). Nonetheless, only few published studies investigated iron metabolism patterns in patients with MG and factors associated with it.

Therefore, in this study, we focused on iron metabolism parameters available in most clinical settings worldwide, including serum iron (SI), ferritin, transferrin (Tf), transferrin saturation (TS), and total iron-binding capacity (TIBC), which are also recommended by many guidelines when iron metabolism disorders are suspected (11). Isolated iron deficiency without inflammation usually features low SI, ferritin, and TS, and elevated Tf and TIBC, while inflammation alone often leads to decreased SI, TS, Tf, and TIBC, and high ferritin (12). Thus, to determine the exact type of iron metabolism disorder, assessing a combination of parameters is necessary.

The primary objectives of the study were to compare iron metabolism patterns between non-anemic immunotherapy-naive patients with MG and healthy controls as well as between the same group of patients with MG before and after receiving immunotherapy, and to identify predictors associated with iron metabolism abnormalities in these patients.

## Materials and methods

The first and second parts of the study were cross-sectional, and the third part involved a longitudinal follow-up. In the first and second parts, baseline iron metabolism parameters of immunotherapy-naive patients with MG were compared with those of matched healthy controls, after which clinical characteristics of these patients were analyzed to identify predictors of aberrant iron metabolism. In the third part of the study, after 12 ± 3 months of immunotherapy, iron metabolism parameters of patients with MG were compared with baseline iron metabolism data of the same group of patients. Reporting of the research adhered to the statement of Strengthening the Reporting of Observational Studies in Epidemiology (STROBE) (13).

### Subjects and outcomes

Our research recruited 105 eligible patients with MG from the Peking Union Medical College Hospital (PUMCH), Beijing, China, from July 2018 to March 2022. The inclusion criteria were as follows: 1) confirmed diagnosis of MG (typical muscle weakness with positive pharmacological testing, electromyography studies, or MG-specific antibody assays) (14), 2) a hemoglobin level ≥ 120 g/L for male or ≥ 110 g/L for female patients, and not on iron supplementary therapy, 3) the minimal manifestation status (MMS) not reached and immunotherapy required to induce the MMS, and 4) no history of receiving immunotherapy, which refers to glucocorticoids and/or immunosuppressants. The exclusion criteria were as follows: 1) comorbidity with clinically diagnosed malignant tumor, 2) comorbidity with evident hepatic [alanine aminotransferase (ALT) or aspartate aminotransferase (AST) > 120 U/L] or renal [estimated glomerular filtration rate (eGFR) < 90 mL·min^−1^·1.73 m^−2^] abnormalities, 3) comorbidity with severe cardiac or hematological diseases, 4) negativity for AChR-Ab and MuSK-Ab with an onset age <13 years, or 5) incomplete clinical data.

To compare iron metabolism patterns of immunotherapy-naive patients with MG with those of the normal population, 105 gender- and age-matched healthy controls who underwent a thorough physical examination in the Department of Health Management, PUMCH, were included. The criteria for a healthy control were as follows: 1) a hemoglobin count ≥ 120 g/L for male or ≥ 110 g/L for female individuals, 2) the absence of evident hepatic (ALT or AST > 120 U/L) or renal (eGFR < 90 mL·min-1·1.73 m^−2^) abnormalities, 3) the absence of severe cardiac or hematological diseases, 4) the absence of clinically suspected malignant tumor, 5) the absence of documented autoimmune diseases or active infection.

**Figure 1:**
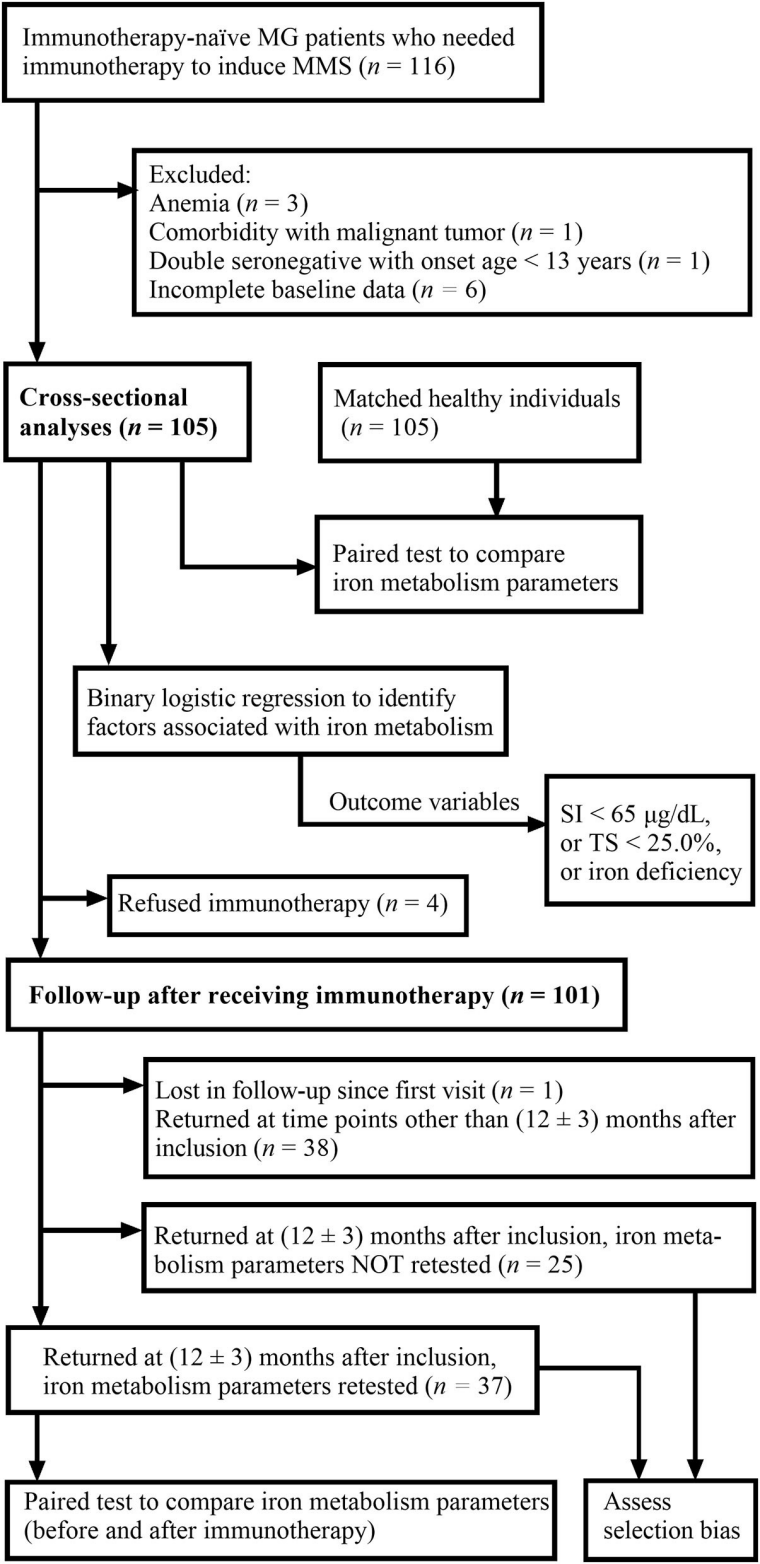
Flowchart demonstrating the process of the study. MG, myasthenia gravis; MMS, minimal manifestation status; SI, serum iron; TS, transferrin saturation.

At baseline, clinical characteristics and iron metabolism parameters of included patients were collected, and cross-sectional analyses were conducted. Then, except for four patients who refused immunotherapy, the remaining 101 patients were started on immunotherapy at inclusion. The patients returned to our MG clinic for follow-up at variable time points, and the follow-up of all patients ended on 30 September 2022. Iron metabolism parameters of the patients who returned at 12 ± 3 months after inclusion were compared with baseline iron metabolism data of the same group of patients. For patients who returned more than one time within 12 ± 3 months, only the follow-up closest to 12 months after inclusion was taken into consideration. In addition, clinical characteristics of patients who returned at our specified time period but were not retested for iron metabolism parameters were compared with clinical characteristics of those who were retested in order to assess potential selection bias. The flowchart in Figure 1 summarizes the whole process. The sample size was not calculated beforehand since few previous data on iron metabolism patterns in MG were present.

Iron metabolism parameters involved were SI (reference range 65–175 μg/dL), Tf (reference range 2.00–3.60 g/L), TIBC (reference range 250–450 μg/dL), TS (reference range 25.0–50.0%), and ferritin (24–336 ng/mL). Reference ranges were determined by the Department of Laboratory Medicine, PUMCH.

Potential confounding variables that may affect iron metabolism were taken into account, which were body mass index (BMI), comorbid diabetes mellitus, comorbid fatty liver disease, use of antithrombotic drugs, heavy alcohol consumption, and mild hepatic or renal insufficiency. Fatty liver disease was diagnosed by abdominal ultrasonography at the Department of Radiology, PUMCH. Antithrombotic drugs included antiplatelet and anticoagulant drugs. Heavy alcohol consumption was defined as a daily intake > 60 g of ethanol for male or > 40 g of ethanol for female individuals in the past year, as derived from the World Health Organization risk drinking levels (15). Mild hepatic insufficiency was defined as a mildly elevated ALT or AST level of 40–120 U/L. Mild renal insufficiency was defined as a serum creatinine level > 104 μmol/L for male or > 84 μmol/L for female individuals, with an eGFR ≥ 90 mL·min-1·1.73 m^−2^.

Since inflammation in MG might distort interpretation of iron deficiency, we defined iron deficiency as ferritin < 100 ng/ml, or TS < 20% if ferritin was 100-299 ng/ml (16), in attempt to detect iron deficiency under inflammatory conditions. Severe generalized disease (SGD) was derived from MG activities of daily living (MG-ADL) scores to dichotomize disease severity. SGD was defined as an MG-ADL score ≥ 6 with at least 1 point in non-ocular items (17). Notably, in our study, we used “iron inadequacy” as the general term for all degrees of iron shortage in plasma since “iron deficiency” already has a specific definition, as mentioned above.

### Statistical analysis

Numerical variables were expressed as mean ± standard deviation if they had a normal distribution, otherwise as median [interquartile range (IQR)]. When comparing two matched samples, paired Student's *t*-test was preferred when both the samples had a normal distribution, otherwise the paired Wilcoxon signed-rank test was used. When comparing two independent samples, Student's *t*-test was preferred when both the samples had a normal distribution, otherwise the Mann–Whitney U test was adopted. For comparison of proportions, the chi-square test was conducted.

To determine factors independently associated with low SI, low TS, and iron deficiency, univariate and multivariate binary logistic regression models were used. Variables with a *p*-value < 0.10 in the univariate analysis were included in the multivariate binary logistic regression. The significance level (α) was set at 0.05 (two-tailed). Odds ratios (ORs) with 95% confidence interval (CI) were displayed, when appropriate. Data analysis was facilitated by SPSS 26 (IBM, NY, USA).

## Results

### Comparison of iron metabolism patterns between patients with MG and controls

A total of 53 male and 52 female patients with MG were recruited for the study. The median age at onset and at inclusion were 51.5 (IQR 32.7–61.4) and 55.4 (IQR 36.6–62.6) years. The median interval between the onset and inclusion was 1.01 (IQR 0.39–3.59) years. Among the 105 patients, 83 were positive for AChR-Ab, six were positive for MuSK-Ab, and 16 were negative for both. As for their MGFA clinical classification at inclusion, 38, 16, 43, and eight of the patients were categorized as MGFA I, II, III, IV and V, respectively; five of the included patients were comorbid with other autoimmune diseases, 20 had thymoma, and 21 had undergone thymectomy before inclusion. The median MG-ADL scores of the patients were 4 (IQR 2–7) points, and 34 patients (32.4%) had SGD.

**Figure 2:**
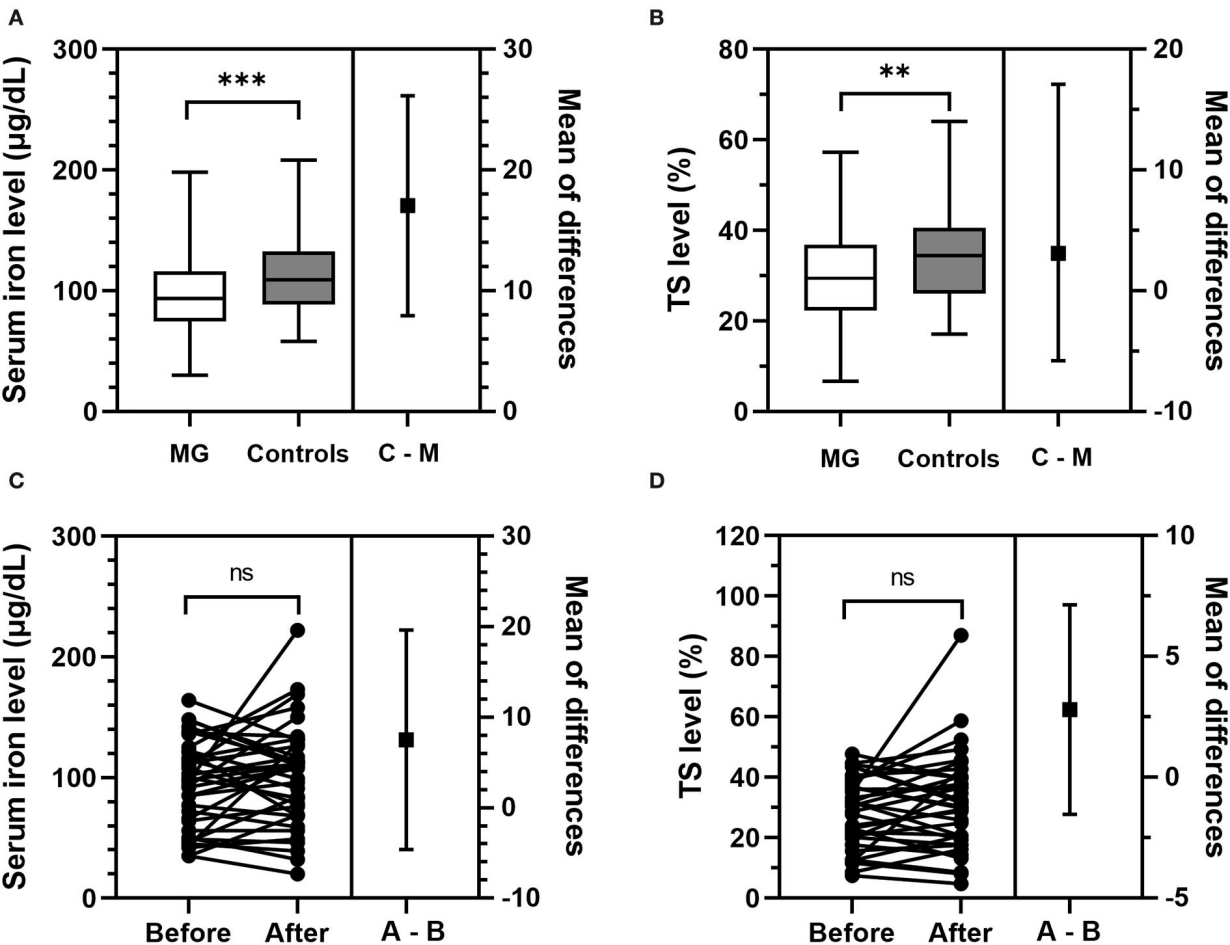
Comparison of SI and TS levels between immunotherapy-naive patients with MG and controls, and between patients with MG before and after initiation of immunotherapy. **(A,B)** Compared with matched healthy controls, immunotherapy-naive patients with MG had significantly lower levels of SI (*p* < 0.001) and TS (*p* = 0.002). “C–M” stands for “controls minus MG,” which is the difference in the SI level or TS level between patients with MG and controls. **(C,D)** The same group of patients with MG had comparable levels of SI (*p* = 0.217) and TS (*p* = 0.420) before and after starting immunotherapy. “A–B” stands for “after minus Before,” which is the difference in the SI level or TS level between patients with MG before and after receiving immunotherapy. ^**^p < 0.01, ^***^p < 0.001. MG, myasthenia gravis; ns, not significant (*p* ≥ 0.05); SI, serum iron; TS, transferrin saturation.

Baseline characteristics and iron metabolism parameters of patients with MG were compared with those of normal controls (Table 1). The MG group and the matched control group were comparable in gender, age at inclusion, time at inclusion, iron deficiency rate, and hemoglobin, Tf, and TIBC levels. Compared with the controls, the patients with MG had significantly lower levels of SI (*p* < 0.001) and TS (*p* = 0.002) (Figures 2A,B), and a significantly larger proportion of the patients with MG had SI and TS levels below the normal range (*p* = 0.001 and *p* = 0.007, respectively) (Table 1). Numerically, the patients with MG had slightly lower hemoglobin and ferritin levels than the controls (*p* = 0.090 and 0.163, respectively), and more patients with MG than controls had Tf and TIBC levels lower than the normal range (*p* = 0.161 and 0.167, respectively), but the effects were not statistically significant.

**Table 1:** Baseline characteristics and iron metabolism parameters of patients with MG and controls.

	**MG patients**	**Controls**	***p*–value**
Female (%)	52/105 (49.5%)	52/105 (49.5%)	1.000
Age at inclusion (years)	55.4 (36.6–62.6)	55.0 (38.3–62.4)	0.788
Difference in age at inclusion (years)	0.05 ± 1.79		N/A
Difference in time at inclusion (months)	−0.13 (−0.52–0.45)		N/A
Hemoglobin (g/L)	145 (133–156)	146 (136–159)	0.090
Serum iron (μg/dL)	95 ± 35	109 (89–133)	< 0.001***
Below normal range (%)	21/105 (20.0%)	4/105 (3.8%)	0.001**
Above normal range (%)	1/105 (1.0%)	2/105 (1.9%)	1.000
Transferrin (g/L)	2.35 (2.09–2.63)	2.35 ± 0.30	0.597
Below normal range (%)	18/105 (17.1%)	11/105 (10.5%)	0.161
Above normal range (%)	0/105 (0.0%)	0/105 (0.0%)	N/A
Total iron–binding capacity (μg/dl)	324 ± 61	321 ± 42	0.721
Below normal range (%)	10/105 (9.5%)	4/105 (3.8%)	0.167
Above normal range (%)	2/105 (1.9%)	0/105 (0.0%)	0.498
Transferrin saturation (%)	29.0 ± 11.7	34.5 ± 10.3	0.002**
Below normal range (%)	37/91 (40.7%)	20/91 (22.0%)	0.007**
Above normal range (%)	5/91 (5.5%)	7/91 (7.7%)	0.550
Ferritin (ng/ml)	88 (41–162)	98 (49–160)	0.163
Below normal range (%)	12/105 (11.4%)	7/105 (6.7%)	0.229
Above normal range (%)	3/105 (2.9%)	2/105 (1.9%)	1.000
Iron deficiency (%)	57/94 (60.6%)	55/94 (58.5%)	0.766

Numbers are displayed as mean ± standard deviation for variables having a normal distribution or median with interquartile range in parentheses for variables not having a normal distribution. **p < 0.01, ***p < 0.001. MG, myasthenia gravis; N/A, not applicable.

In addition, the patients with MG and healthy controls were similar with regard to BMI, comorbid diabetes mellitus, comorbid fatty liver disease, use of antithrombotic drugs, heavy alcohol consumption, and mild hepatic or renal insufficiency (Supplementary Table S1), which meant our results were probably not affected by these confounders. The median hypersensitive C-reactive protein level in the healthy subjects was 0.78 (0.38–1.29) mg/L, ruling out acute inflammation in them.

### Factors associated with iron metabolism pattern in MG

Since our cross-sectional analyses revealed that the immunotherapy-naive patients with MG had significantly lower SI and TS levels than the healthy controls, we were interested to identify whether any clinical variables were associated with decreased SI and TS levels in the patients with MG, and we also took iron deficiency into account. Thus, univariate and multivariate binary logistic regression models were adopted to identify potential predictors of low SI (SI < 65 μg/dL), low TS (TS < 25.0%), and iron deficiency.

Since menstruation may affect iron metabolism, we first compared hemoglobin and iron metabolism parameters among premenopausal female, postmenopausal female, and male patients with MG (Table 2). Indeed, the premenopausal female patients had significantly lower levels of hemoglobin (*p* < 0.001) and ferritin (*p* < 0.001) than male patients, and a significantly larger proportion of the premenopausal female patients had an SI level below the normal range (*p* = 0.023) and iron deficiency (*p* < 0.001). By contrast, the postmenopausal female patients had similar iron metabolism parameters to the male patients (*p* ≥ 0.05), although the postmenopausal female patients had significantly lower hemoglobin levels (*p* < 0.001). To further explore its role in female patients with MG, menstruation was also taken into account as a predictor variable in the following analyses.

**Table 2:** Comparison of iron metabolism parameters among premenopausal female, postmenopausal female, and male patients with MG.

	**PreM F**	**PostM F**	**Male**	***p*_1_**	***p*_2_**	***p*_3_**
Hb (g/L)	136 ± 10	135 ± 10	153 ± 12	1.000	< 0.001***	< 0.001***
SI (μg/dL)	92 ± 45	88 ± 34	99 ± 28	0.978	0.850	0.384
Below normal (%)	9/25 (36.0%)	6/27 (22.2%)	6/53 (11.3%)	0.273	0.023*	0.337
Tf (g/L)	2.51 ± 0.37	2.36 ± 0.47	2.31 ± 0.40	0.580	0.160	1.000
TIBC (μg/dl)	344 ± 56	318 ± 72	317 ± 55	0.364	0.190	1.000
TS (%)	26.9 ± 13.9	27.3 ± 11.0	31.1 ± 9.8	1.000	0.377	0.476
Below normal (%)	13/25 (52.0%)	12/27 (44.4%)	17/53 (32.1%)	0.586	0.091	0.277
Ferritin (ng/ml)	33 (15–56)	97 (40–155)	121 (78–212)	0.002**	< 0.001***	0.105
Iron deficiency (%)	24/25 (96.0%)	16/27 (59.3%)	21/53 (39.6%)	0.002**	< 0.001***	0.096

Numbers are displayed as mean ± standard deviation for variables having a normal distribution or median with interquartile range in parentheses for variables not having a normal distribution. *p < 0.05, **p < 0.01, ***p < 0.001. Hb, hemoglobin; MG, myasthenia gravis; PostM F, postmenopausal female; PreM F, premenopausal female; *p*_1_, the *p*–value between PreM F and PostM F; *p*_2_, the *p*–value between PreM F and male; *p*_3_, the *p*–value between PostM F and male; SI, serum iron; Tf, transferrin; TIBC, total iron–binding capacity; TS, transferrin saturation.

Among the 105 immunotherapy-naive patients with MG, 21 (20.0%) had low SI, and the number of patients with low TS was 42 (40.0%). Predictor variables included in the univariate binary logistic regression model were gender and menstruation status (premenopausal female vs. others, and postmenopausal female vs. others), onset age (≥ vs. < 50 years old), disease course (≥ vs. < 1 year), antibody status (MuSK-Ab vs. others), SGD at inclusion (yes vs. no), comorbidity with thymoma (yes vs. no), history of thymectomy (yes vs. no), and comorbidity with other autoimmune diseases (yes vs. no). In univariate analyses, premenopausal female (*p* = 0.026) was the only predictor found to be significantly associated with SI < 65 μg/dL, with a *p-*value < 0.10 and also < 0.05. None of the predictor variables significantly correlated with TS < 25.0% (*p* ≥ 0.05). In the univariate analyses, premenopausal female (*p* = 0.001) and SGD (*p* = 0.017) were potentially associated with iron deficiency, with a *p*-value < 0.10, and were thus included in the multivariate model. After adjustment, premenopausal female (*p* = 0.002) was still significantly associated with iron deficiency, while SGD was not. To be specific, the non-anemic immunotherapy-naive patients with MG who were premenopausal female were approximately three times more likely to have an SI level < 65 μg/dL and 24 times more likely to suffered iron deficiency than the other patients (Table 3).

**Table 3:** Potential predictors of low SI or iron deficiency.

**Predictor variables**	**Outcome variables**	**Univariate analysis**		**Multivariate analysis**	
		**Crude OR (95% CI)**	***p***	**Adjusted OR (95% CI)**	***p***
Premenopausal female vs. others	Low SI	3.187 (1.148–8.853)	0.026*	N/A	N/A
	ID	27.892 (3.598–216.248)	0.001**	24.014 (3.067–188.018)	0.002**
Postmenopausal female vs. others	Low SI	1.200 (0.413–3.491)	0.738	N/A	N/A
	ID	1.067 (0.438–2.596)	0.887	N/A	N/A
Onset age ≥ 50 years old (yes vs. no)	Low SI	0.590 (0.225–1.551)	0.285	N/A	N/A
	ID	0.571 (0.260–1.255)	0.163	N/A	N/A
Positive MuSK–Ab (yes vs. no)	Low SI	2.105 (0.359–12.354)	0.410	N/A	N/A
	ID	0.707 (0.136–3.679)	0.680	N/A	N/A
Disease course ≥ 1 year (yes vs. no)	Low SI	0.867 (0.333–2.258)	0.770	N/A	N/A
	ID	0.550 (0.251–1.205)	0.135	N/A	N/A
SGD at inclusion (yes vs. no)	Low SI	0.800 (0.280–2.286)	0.677	N/A	N/A
	ID	0.357 (0.154–0.829)	0.017*	0.509 (0.206–1.262)	0.145
History of thymoma (yes vs. no)	Low SI	1.437 (0.456–4.535)	0.536	N/A	N/A
	ID	0.519 (0.194–1.388)	0.191	N/A	N/A
History of thymectomy (yes vs. no)	Low SI	1.289 (0.411–4.043)	0.663	N/A	N/A
	ID	0.480 (0.182–1.268)	0.139	N/A	N/A
Comorbid autoimmune diseases (yes vs. no)	Low SI	1.000 (0.106–9.444)	1.000	N/A	N/A
	ID	3.018 (0.326–27.973)	0.331	N/A	N/A

Univariate and multivariate binary logistic regression models were adopted, and predictor variables with a *p*–value < 0.10 in the univariate analysis were included in the multivariate binary logistic regression. *p < 0.05, **p < 0.01. CI, confidence interval; ID, iron deficiency; MuSK–Ab, anti–muscle–specific tyrosine kinase antibody; N/A, not applicable; OR, odds ratio; SGD, severe generalized disease; SI, serum iron.

### Comparison of iron metabolism pattern in MG before and after immunotherapy

We then took one step further and explored whether iron metabolism characteristics of the immunotherapy-naive patients with MG altered after receiving immunotherapy for a specific period of time. Among the 105 patients, 101 were started on immunotherapy at inclusion, and 100 patients returned to the hospital for follow-up at variable time points, among whom 62 came back at 12 ± 3 months after initiating immunotherapy, and 37 of them were again tested for iron metabolism parameters, while the other 25 patients refused to be tested again.

After receiving immunotherapy for 12 ± 3 months, hemoglobin, SI, Tf, TIBC, TS, and ferritin levels, as well as the iron deficiency rate of the 37 patients were all comparable (*p* ≥ 0.05) with those of the same group patients before initiation of immunotherapy (Table 4, Figures 2C,D). The ratio of patients with abnormal iron metabolism parameters did not differ significantly before and after immunotherapy as well (*p* ≥ 0.05).

**Table 4:** Iron metabolism parameters of patients with MG before and after initiation of immunotherapy.

	**Before immunotherapy**	**After immunotherapy**	***p*–value**
Hemoglobin (g/L)	143 ± 16	141 ± 15	0.262
Serum iron (μg/dL)	94 ± 36	102 ± 43	0.217
Below normal range (%)	10/37 (27.0%)	7/37 (18.9%)	0.407
Above normal range (%)	0/37 (0.0%)	1/37 (2.7%)	1.000
Transferrin (g/L)	2.40 ± 0.37	2.41 ± 0.37	0.827
Below normal range (%)	4/37 (10.8%)	4/37 (10.8%)	1.000
Above normal range (%)	0/37 (0.0%)	0/37 (0.0%)	N/A
Total iron–binding capacity (μg/dl)	323 ± 54	336 ± 55	0.107
Below normal range (%)	2/37 (5.4%)	1/37 (2.7%)	1.000
Above normal range (%)	0/37 (0.0%)	1/37 (2.7%)	1.000
Transferrin saturation (%)	28.6 ± 11.6	30.4 (20.0–40.1)	0.420
Below normal range (%)	16/37 (43.2%)	13/37 (35.1%)	0.475
Above normal range (%)	0/37 (0.0%)	3/37 (8.1%)	0.240
Ferritin (ng/ml)	73 (34–167)	76 (26–155)	0.862
Below normal range (%)	5/37 (13.5%)	9/37 (24.3%)	0.235
Above normal range (%)	1/37 (2.7%)	2/37 (5.4%)	1.000
Iron deficiency (%)	24/37 (64.9%)	23/37 (62.2%)	0.809

Numbers are displayed as mean ± standard deviation for variables having a normal distribution or median with interquartile range in parentheses for variables not having a normal distribution. MG, myasthenia gravis; N/A, not applicable.

Since only 37 of the 62 patients (59.7%) returning at 12 ± 3 months after inclusion had iron metabolism parameters retested, we were concerned whether any selection bias would exist. Thus, we then compared clinical characteristics of patients who did and did not take retest for iron metabolism parameters, and it turned out that no significant differences were detected between the two groups of patients (*p* ≥ 0.05), suggesting that the two groups were comparable with regard to those characteristics and that our previous results were probably not interfered by selection bias (Supplementary Table S2).

## Discussion

In this study, we hypothesized that iron metabolism patterns of the immunotherapy-naive patients with MG were different from those of the healthy individuals and might alter after starting immunotherapy. Key findings were as follows: 1) compared with the healthy individuals, the non-anemic immunotherapy-naive patients with MG had significantly lower levels of SI and TS, and a significantly larger proportion of the patients with MG had SI and TS levels below the normal range; 2) the premenopausal female patients have an SI level < 65 μg/dL and iron deficiency, but not TS < 25.0% in these patients with MG; and 3) iron metabolism parameters did not alter significantly after 12 ± 3 months of immunotherapy in patients with MG.

### Iron metabolism in immunotherapy-naive patients with MG

Both iron inadequacy and inflammation affect iron metabolism. SI and TS levels decrease in iron inadequacy and inflammation. In iron inadequacy, ferritin is reduced, while Tf and TIBC increased. By contrast, the situation is quite the opposite in inflammation because ferritin and transferrin are, respectively, positive and negative acute-phase protein (18, 19), and TIBC is dependent on the concentration of Tf (20).

Our results showed that the immunotherapy-naive patients with MG had lower SI and TS levels than the healthy individuals, but they had similar Tf, TIBC, and ferritin levels to the healthy individuals, which indicated that iron inadequacy and inflammation coexisted in the patients with MG because when iron inadequacy and inflammation coexist, both of them would lead to decreased SI and TS, while their effects on Tf, TIBC, and ferritin levels might be offset by each other. Although inflammation in an autoimmune disease like MG seemed to be self-evident, we demonstrated that the non-anemic immunotherapy-naive patients with MG also suffered iron inadequacy, which might have resulted from both insufficient iron intake and chronic inflammatory status. Common symptoms of iron inadequacy include fatigue and lethargy (18), which, when complicated with MG, might be attributed by clinicians to MG, instead of iron inadequacy, leading to underdiagnosis of iron inadequacy in such circumstances. Thus, it is recommended to take iron inadequacy into consideration in the patients with MG complaining fatigue, lethargy, or similar symptoms.

Approaches for adjusting ferritin to reflect “true iron inadequacy” are required in the presence of inflammation. Based on previous studies, we defined iron deficiency as ferritin < 100 ng/ml, or TS < 20% if ferritin was 100–299 ng/ml (16). Nevertheless, based on this definition, we found that the prevalence of iron deficiency was similar between patients with MG and healthy controls, which might hint that this definition of iron deficiency was still obscured by inflammation in the patients with MG, since our previous analyses already suggested that iron inadequacy was present in MG.

### Factors associated with iron metabolism in MG

Premenopausal female patients are especially vulnerable to iron inadequacy and may suffer severe health consequences if not diagnosed and treated properly (21). Indeed, in the immunotherapy-naive patients with MG, we found that premenopausal female patients had a low SI level and iron deficiency, and the positive correlation between the premenopausal female patients and iron deficiency remained significant after adjustment, which showed more prominent iron inadequacy in premenopausal female than male patients with MG. In multiple sclerosis (MS), another autoimmune disease of the nervous system, van Rensburg et al. found that Caucasian female patients with MS had significantly lower serum iron and ferritin concentrations than matched controls (7), which was partly in accordance with our results.

Counter-intuitively, our findings in univariate logistic regression revealed that SGD seemed to be a protective factor for “iron deficiency,” which, however, might actually result from more severe inflammation, instead of milder iron inadequacy in the patients with MG with SGD. As mentioned earlier, our criteria for iron deficiency might still be obscured by inflammation in MG. Since SGD was not shown to be a protective factor for SI or TS in our patients, iron inadequacy was unlikely to be milder in patients with SGD than in non-SGD patients. Instead, the negative correlation of SGD with “iron deficiency” might be attributed to more severe inflammation in patients with SGD, which led to elevated ferritin levels and masked true iron inadequacy under such circumstances. Our previous observation showed that the ferritin level was independently associated with SGD in immunotherapy-naive patients with MG (unpublished data), which also supported the aforementioned assumption. After adjustment using multivariate logistic regression, SGD was no longer significantly associated with iron deficiency.

At 12 ± 3 months of follow-up, although we noticed that hemoglobin, SI, and TS levels of the patients with MG after immunotherapy were slightly different from those before therapy, hemoglobin and iron metabolism parameters were statistically comparable with those at inclusion, which suggested no explicit evidence of alleviation or worsening of iron inadequacy after treatment. Indeed, we do not assume iron inadequacy in patients with MG would alleviate spontaneously without iron supplementation because drugs commonly used in MG, including glucocorticoids, proton pump inhibitors, and histamine-2 receptor antagonists, may, on the contrary, aggravate iron inadequacy (22, 23). We also found that clinical characteristics were similar between the patients who did and did not take retest for iron metabolism parameters when they came back at 12 ± 3 months after inclusion, which helped rule out selection bias.

### Limitations and highlights

The limitation of this study lies in its relatively small sample size, which might only be able to detect relatively obvious effects. In addition, since the soluble transferrin receptor (sTfR) and sTfR–ferritin index were only tested in a small number of included patients, they were not analyzed.

However, our study is novel in its kind and is probably the first study to analyze the iron metabolism pattern and factors associated with it in patients with MG, and to compare the iron metabolism pattern between patients with MG and healthy individuals, and that in the same group of patients before and after immunotherapy. Our study also implied that testing iron metabolism parameters and subsequent iron supplementation therapy (e.g., oral iron supplements) for eligible patients with MG might be justifiable since iron inadequacy is unlikely to improve without iron supplementation after immunotherapy, as discussed previously (22, 23). Moreover, according to recent guidelines, oral iron supplementation is recommended in iron inadequacy associated with chronic diseases (12). Although no consensus has been reached on treatment targets, it seems reasonable to sustain iron supplementation until ferritin and TS return to normal ranges (11).

To further study the pattern and mechanism of iron metabolism disorders in MG, future research should consider investigating other important parameters, including sTfR, sTfR/log ferritin index (24), hepcidin, interleukin-6 (25), and free erythrocyte protoporphyrin. It is also recommended to conduct large prospective studies to explore the indications for iron supplementation therapy in iron-inadequate patients with MG, and to compare clinical outcomes among those patients with MG with different regimes of and without iron supplementation. Feasible outcome variables might include clinical assessments [MG-ADL, MG quality of life (MG-QOL), etc.] and iron metabolism parameters (ferritin, TS, SI, etc.).

## Conclusion

Non-anemic immunotherapy-naive patients with MG had significantly lower levels of SI and TS than healthy individuals. Premenopausal female was significantly associated with SI < 65 μg/dL and iron deficiency in these patients. Iron metabolism parameters did not significantly alter after around 12 months of immunotherapy in the same group of patients. Iron inadequacy in patients with MG, particularly premenopausal female patients, should be given more importance, given the essential role of iron in multiple physiological and pathophysiological processes.

## Data availability statement

The raw data supporting the conclusions of this article will be made available by the authors, without undue reservation.

## Ethics statement

The studies involving human participants were reviewed and approved by Ethics Committee of Clinical Research of Peking Union Medical College Hospital, Beijing, China. Written informed consent to participate in this study was provided by the participants' legal guardian/next of kin.

## Author contributions

KL: conceptualization (equal), data curation (lead), formal analysis (lead), investigation (lead), methodology (equal), software (lead), visualization (lead), and writing—original draft (lead). LH: data curation (lead), formal analysis (equal), investigation (equal), methodology (equal), resources (equal), validation (equal), and writing—review and editing (equal). YT: data curation (equal), formal analysis (equal), investigation (equal), methodology (lead), resources (equal), validation (equal), and writing—review and editing (equal). YH, JS, JH, and JY: data curation (equal), formal analysis (equal), investigation (equal), methodology (equal), resources (equal), validation (equal), and writing—review and editing (equal). YG: conceptualization (lead), data curation (lead), formal analysis (equal), funding acquisition (lead), investigation (equal), methodology (equal), project administration (lead), resources (lead), supervision (lead), validation (lead), and writing—review and editing (lead). All authors contributed to the article and approved the submitted version.

## Funding

This work was supported by the Non-profit Central Research Institute Fund of Chinese Academy of Medical Sciences (grant number: 2019XK320039) and the National High Level Hospital Clinical Research Funding (grant number: 2022-PUMCH-B-017).

## Conflict of interest

The authors declare that the research was conducted in the absence of any commercial or financial relationships that could be construed as a potential conflict of interest.

## Publisher's note

All claims expressed in this article are solely those of the authors and do not necessarily represent those of their affiliated organizations, or those of the publisher, the editors and the reviewers. Any product that may be evaluated in this article, or claim that may be made by its manufacturer, is not guaranteed or endorsed by the publisher.
