# Supplementary material for: Iron metabolism patterns in non-anemic patients with myasthenia gravis: A cross-sectional and follow-up study
Source: Front Neurol. 2022 Nov 24;13:1060204. doi: 10.3389/fneur.2022.1060204 (PMC9729274; doi:10.3389/fneur.2022.1060204)
Supplement: Supplementary file 1 [file Table_1.docx]

**Supplementary Table S1** Potential confounding variables for iron metabolism MG patients and controls.

|  | MG patients | Controls | *p* value |
| --- | --- | --- | --- |
| BMI (kg/m^2^) | 25.30 ± 4.28 | 24.32 ± 3.38 | 0.204 |
| Comorbid diabetes mellitus (%) | 16/105 (15.2%) | 14/105 (13.3%) | 0.693 |
| Comorbid fatty liver disease (%) | 4/11 (36.4%) | 49/105 (46.7%) | 0.514 |
| Use of antithrombotic drugs (%) | 2/105 (1.9%) | 3/105 (2.9%) | 1.000 |
| Heavy alcohol consumption (%) | 2/105 (1.9%) | 0/105 (1.9%) | 0.498 |
| Mildly elevated ALT (%) | 3/105 (2.9%) | 7/105 (6.7%) | 0.195 |
| Mildly elevated AST (%) | 0/105 (1.9%) | 1/105 (1.0%) | 1.000 |
| Mild renal insufficiency (%) | 0/105 (1.9%) | 1/105 (1.0%) | 1.000 |

Numbers are displayed as mean ± standard deviation for variables having a normal distribution or median with interquartile range in parentheses for variables not having a normal distribution. Note that only 11 MG patients underwent abdominal ultrasonography to assess fatty liver disease. Mildly elevated ALT or AST was defined as a ALT or AST level of 40-120 U/L. Mild renal insufficiency was defined as serum creatinine > 104 μmol/L for male or > 84 μmol/L for female, with eGFR ≥ 90 mL·min-1·1.73 m^-2^. ^*^*p* < 0.05. ALT, alanine aminotransferase; AST, aspartate aminotransferase; eGFR, estimated glomerular filtration rate; MG, myasthenia gravis.
