# Supplementary material for: Iron metabolism patterns in non-anemic patients with myasthenia gravis: A cross-sectional and follow-up study
Source: Front Neurol. 2022 Nov 24;13:1060204. doi: 10.3389/fneur.2022.1060204 (PMC9729274; doi:10.3389/fneur.2022.1060204)
Supplement: Supplementary file 2 [file Table_2.docx]

**Supplementary Table S2** Clinical characteristics of MG patients 12 ± 3 months after starting immunotherapy whose iron metabolism parameters were and were not retested.

|  | Iron metabolism parameters retested | Iron metabolism parameters NOT retested | *p*-value |
| --- | --- | --- | --- |
| Female (%) | 20/37 (54.1%) | 11/25 (44.0%) | 0.437 |
| Age at onset (years) | 46.2 ± 18.6 | 50.5 ± 16.4 | 0.351 |
| Positive MuSK-Ab (%) | 3/37 (8.1%) | 1/25 (4.0%) | 0.642 |
| History of thymoma (%) | 7/37 (18.9%) | 5/25 (20.0%) | 0.916 |
| History of thymectomy (%) | 7/37 (18.9%) | 5/25 (20.0%) | 0.916 |
| Comorbid autoimmune diseases (%) | 2/37 (5.4%) | 2/25 (8.0%) | 1.000 |
| Disease course at inclusion (years) | 0.97 (0.43-3.49) | 1.01 (0.23-3.57) | 0.482 |
| Hemoglobin at inclusion (g/L) | 143 ± 16 | 144 ± 14 | 0.847 |
| Serum iron at inclusion (μg/dL) | 94 ± 36 | 90 ± 32 | 0.690 |
| Below normal range (%) | 10/37 (27.0%) | 5/25 (20.0%) | 0.526 |
| Transferrin at inclusion (g/L) | 2.40 ± 0.37 | 2.31 ± 0.38 | 0.375 |
| TIBC at inclusion (μg/dl) | 323 ± 54 | 314 ± 61 | 0.541 |
| Transferrin saturation at inclusion (%) | 28.6 ± 11.6 | 28.5 ± 11.2 | 0.973 |
| Below normal range (%) | 16/37 (43.2%) | 11/25 (44.0%) | 0.953 |
| Ferritin at inclusion (ng/ml) | 73 (34-167) | 99 (71-185) | 0.173 |
| Iron deficiency at inclusion (%) | 24/37 (64.9%) | 14/25 (56.0%) | 0.482 |
| MG-ADL at inclusion (points) | 3 (2-7) | 4 (2-8) | 0.213 |
| SGD at inclusion (%) | 10/37 (27.0%) | 9/25 (36.0%) | 0.452 |
| MG-ADL at follow-up (points) | 0 (0-1) | 0 (0-2) | 0.104 |

Numbers are displayed as mean ± standard deviation for variables having a normal distribution or median with interquartile range in parentheses for variables not having a normal distribution. ^*^*p* < 0.05. MG, myasthenia gravis; MG-ADL, MG activities of daily living; MuSK-Ab, anti-muscle-specific tyrosine kinase antibody; SGD, severe generalized disease; TIBC, total iron-binding capacity.
